# Heat shock response enhanced by cell culture treatment in mouse embryonic stem cell-derived proliferating neural stem cells

**DOI:** 10.1371/journal.pone.0249954

**Published:** 2021-04-14

**Authors:** Hiroyuki Omori, Masahiro Otsu, Haruo Nogami, Masayoshi Shibata

**Affiliations:** 1 Department of Physical Therapy, School of Health Sciences, Japan University of Health Sciences, Saitama, Japan; 2 Braizon Therapeutics, Inc., Tokyo, Japan; 3 Course of Physical Therapy, Department of Rehabilitation, Faculty of Health Sciences, University of Human Arts and Sciences, Saitama, Japan; Sechenov First Medical University, RUSSIAN FEDERATION

## Abstract

Cells have a regulatory mechanism known as heat shock (HS) response, which induces the expression of HS genes and proteins in response to heat and other cellular stresses. Exposure to moderate HS results in beneficial effects, such as thermotolerance and promotes survival, whereas excessive HS causes cell death. The effect of HS on cells depends on both exogenous factors, including the temperature and duration of heat application, and endogenous factors, such as the degree of cell differentiation. Neural stem cells (NSCs) can self-renew and differentiate into neurons and glial cells, but the changes in the HS response of symmetrically proliferating NSCs in culture are unclear. We evaluated the HS response of homogeneous proliferating NSCs derived from mouse embryonic stem cells during the proliferative phase and its effect on survival and cell death *in vitro*. The number of adherent cells and the expression ratios of HS protein (*Hsp*)*40* and *Hsp70* genes after exposure to HS for 20 min at temperatures above 43°C significantly increased with the extension of the culture period before exposure to HS. In contrast, caspase activity was significantly decreased by extension of the culture period before exposure to HS and suppressed the decrease in cell viability. These results suggest that the culture period before HS remarkably affects the HS response, influencing the expression of HS genes and cell survival of proliferating NSCs in culture.

## Introduction

Cells possess a regulatory mechanism, termed heat shock (HS) response, which allows cells to respond to heat and other cellular stresses. This response includes the induction of expression of HS proteins (HSPs), including Hsp27, Hsp40, Hsp70, and Hsp90 [1, 2]. HS responses in cells under stress are extremely dependent on external factors, including the extent and duration of the temperature elevation and the timing of exposure, with exposure to excess stress potentially inducing cell death. For example, exposure of rat primary cortical and hippocampal neurons to HS at 44 or 45°C for 30 min can induce apoptotic cell death [3]. In contrast, exposure to moderate HS stress enhances thermotolerance by inducing HSP expression, which promotes cell survival. HSP expression induced by moderate heat exposure has been found to have a neuroprotective effect in cultured hippocampal neurons vulnerable to glutamate [4]. Other studies performed under different HS conditions found that prior exposure to mild heat stress protects primary neurons from more severe stress [5, 6].

HS responses also depend on internal factors, including the degree of neural cell differentiation. In the central nervous system, neural stem cells (NSCs) divide symmetrically until a sufficient number is achieved; then, they start to divide asymmetrically to produce neurons, astrocytes, and oligodendrocytes [7, 8]. NSCs are not only observed in neural tissues during early development, but can also be observed in some regions of the fetal and adult brains [9]. Isolated NSCs can be grown *in vitro* in medium supplemented with epidermal growth factor and/or fibroblast growth factor 2 (FGF2), forming spherical cell clusters known as neurospheres [10]. Although previous studies have examined the *in vivo* and *in vitro* effects of HS on the properties of NSCs and neural stem/precursor cells (NS/PCs), how the effects of HS vary depending on the time point of NSCs proliferation and differentiation is unknown. Previous studies have shown that exposure of NSCs to high temperatures during the proliferation phase in the early development arrested cell proliferation and induced cell death in cultured guinea pig [11–13], rat [14], and mouse embryos *in vivo* [15]. Moreover, severe heat stress exposure of NPCs reduced the cell viability of neurospheres [16], whereas mild heat exposure increased NS/PC proliferation in heat-acclimated rats [17]. These findings suggest that exposure to HS affects the survival and proliferation of NSCs and NS/PCs, both *in vivo* and *in vitro*. Although these *in vivo* studies tested the effects of HS on NSCs during the transition from symmetric to asymmetric division, and *in vitro* studies have tested the effects of HS on the properties of NS/PCs in neurospheres, none have examined the effects of HS specifically on symmetrically dividing NSCs in culture.

NSCs can be derived from pluripotent stem cells, such as embryonic stem (ES) cells [18–23] and induced pluripotent stem cells [24–26], and serve as a source of transplantation therapy for some central nervous system disorders. In particular, the neural stem sphere (NSS) method is a simple and efficient method for generating large numbers of homogeneous multipotent NSCs from mouse ES cells [27, 28], which can differentiate into neurons and glia [28–30]. In addition, NSCs prepared using the NSS method are able to maintain their properties in adherent monolayer cultures in the presence of FGF2, making them a suitable *in vitro* model to address the direct effects of exposure to HS [31] and other external stresses [32, 33] on proliferating NSCs. Therefore, in the current study, we evaluated the effects of brief exposure to HS on the response of proliferating NSCs derived from mouse ES cells and the resulting cell survival in different culture periods. We found that the culture period of proliferating NSCs prior to exposure to HS significantly altered the HS response, including HSP expression, altering both cell survival and cell death, which provides insight for determining the effective degree of exposure to HS to NSCs for cell therapy.

## Materials and methods

### Preparation of NSCs using the NSS method

**Fig 1:**
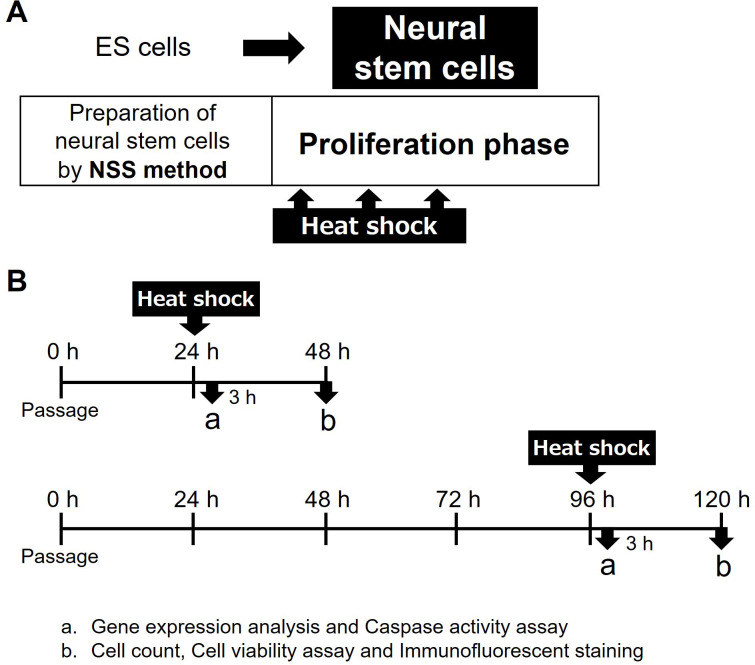
Experimental design and preparation of neural stem cells by the neural stem sphere (NSS) method. (A) Preparation of mouse neural stem cells (NSCs) by NSS method and the times of exposure to heat shock (HS). Large numbers of NSCs were produced by the NSS method directly from mouse ES cells. These cells were cultured on dishes as monolayers and expanded in medium supplemented with FGF2. NSCs in the proliferation phase were exposed to HS. (B) Experimental schedule. NSCs were exposed to HS by immersing the culture dishes in thermostat baths at 37°C, 42°C, 43°C or 44°C after culture at 37°C under an atmosphere of 5% CO_2_ for 24 h (day 0) and 96 h (day 3). 3 h after HS, gene expression and caspase activities were assessed; after 24 h, the numbers of adherent cells, cell viability were measured and immunofluorescence staining was performed.

Mouse NSCs were prepared from undifferentiated mouse ES cells using the NSS method (Fig 1A), as previously described [28, 29, 31]. The mouse ES cell line (Cell No. RBRC-AES0140) was provided by the RIKEN BRC through the National Bio-Resource Project of MEXT, Japan. NSSs were formed by culturing the cells in neuron culture medium (FUJIFILM Wako, Tokyo, Japan) under free-floating conditions. Adherent cells were cultured in the presence of 20 ng/mL FGF2 (R&D Systems, Minneapolis, MN), resulting in large numbers of homogeneous NSCs migrating from the NSSs. The migrating NSCs were collected using conventional trypsin digestion to remove attached NSSs and plated in the presence of FGF2. The proliferating NSCs were suspended in culture media containing 10% dimethyl sulfoxide and stored at -80°C or in liquid nitrogen.

### NSC culture

To prepare NSCs, the cryopreserved cells were thawed and expanded on proliferative monolayer cultures in NSC medium, consisting of neurobasal medium (Gibco, Carlsbad, CA) augmented with 2% B27 supplement (Gibco) and 20 ng/mL FGF2 (Fig 1A). NSCs were cultured at 37°C and 5% CO_2_, and the culture medium was replaced every 48 h.

### Exposure to HS

NSCs were cultured in NSC medium on 35-mm culture dishes (BD Falcon; Becton Dickinson, Franklin Lakes, NJ, USA) for 24 h (time point considered day 0) or 96 h (day 3), tightly sealed with parafilm, and exposed to HS by immersion in thermostat baths (Thermominder TAITEC Co. Ltd., Saitama, Japan) maintained at 37°C, 42°C, 43°C, or 44°C for 20 min (Fig 1B). The cells were allowed to recover by culturing at 37°C and 5% CO_2_ for 24 h.

### Measurement of cell numbers after HS

After the recovery period of 24 h, the number of adherent cells on culture dishes was counted in five cell-containing areas per dish using an inverted microscope equipped with a phase-contrast objective (DIAPHOT; Nikon, Tokyo, Japan).

### Quantitative real-time RT-PCR analysis

After the recovery period of 3 h, mRNA was extracted from NSCs after HS at 24 h and 96 h, along with cells exposed to 37°C. mRNA extraction was performed using the QuickPrep™ Micro mRNA Purification Kit (GE Healthcare Bio-Sciences Corp., NJ, USA), according to the manufacturer’s instructions, although the DNase treatment step was not applied. Changes in the levels of gene expression were determined using real-time RT-PCR analysis with a 7300 Real-Time PCR System (Applied Biosystems, Foster City, CA, USA), Power SYBR^®^GREEN PCR Master Mix (Applied Biosystems), and the corresponding primers. The primer pairs were designed using Primer Express software (Applied Biosystems). Their details are mentioned in Tables 1 and S1. The potential reference genes glyceraldehyde-3-phosphate dehydrogenase (*GAPDH)* and ribosomal protein *S29 (RPS29)* were compared within the reference gene ranking [34]. As *RPS29* expression showed less change after HS, it was chosen as the reference gene. The standard curve method was used to calculate the relative expression of each target gene. Expression values of each gene were normalized relative to the expression levels of *RPS29*.

**Table 1:** Primers used in quantitative real-time RT-PCR.

Symbols	Gene Names	Synonyms	GenBank Accession Numbers
*Gapdh*	glyceraldehydes-3-phosphate dehydrogenase	*Gapd*	NM_008084
*Rps29*	ribosomal protein S29	*S29 ribosomal protein*	NM_009093
*Hspa1b*	heat shock protein 1B	*Hsp70*	NM_010478
*Dnajb1*	DnaJ heat shock protein family (Hsp40) member B1	*Hsp40*	NM_018808
*Hsp90aa1*	heat shock protein 90, alpha (cytosolic), class A member 1	*Hsp90*	NM_010480
*Hspb2*	heat shock protein 2	*Hsp27*	NM_024441
*Cycs*	cytochrome c, somatic	*Cytochrome c*	NM_007808
*Apaf-1*	apoptotic peptidase activating factor 1	*Apaf-1*	NM_009684
*Casp9*	caspase 9	*Caspase-9*	NM_015733
*Casp3*	caspase 3	*Caspase-3*	NM_009810
*Casp7*	caspase 7	*Caspase-7*	NM_007611
*Nes*	nestin	*ESTM46*	NM_016701

### Cell viability assay

After NSCs were exposed to HS at 43°C or 44°C for 20 min and allowed to recover for 24 h at 37°C, along with cells exposed to 37°C, the cell viabilities were assessed. The cells were lysed in lysis buffer (Promega, Madison, WI, USA) for 5 min and were subjected to luminescence-based assays using CellTiter Glo reagent (Promega) according to the manufacturer’s instructions. Luminescence intensities were quantified using a luminometer (TD-20e; Turner Biosystems). The ratio of cell viability after HS was calculated relative to 37°C.

### Caspase activity assay

NSCs were exposed to HS at 43°C or 44°C for 20 min and allowed to recover for 3 h at 37°C. Afterwards, HS-exposed cells along with cells exposed to 37°C were incubated with lysis buffer (Promega, Madison, WI) for 5 min and were subjected to luminescence-based assays with CellTiter Glo reagent (Promega) to assess cell viability. Luminescence intensities were quantified using a luminometer (TD-20e; Turner Biosystems). To assess caspase 3/7 activity, cells were incubated with Caspase-Glo 3/7 reagent (Promega), according to the manufacturer’s instructions. Caspase activity levels were normalized by cell viability levels. The ratio of caspase activity after HS was calculated relative to 37°C.

### Immunofluorescence analysis

NSCs exposed to HS and subsequently allowed to recover, along with cells exposed to 37°C, were evaluated using immunofluorescence staining. Cells were fixed with 4% paraformaldehyde in phosphate-buffered saline (PBS) at room temperature, treated with 0.5% Triton X-100 in PBS, blocked with 10% bovine serum albumin (BSA) in PBS for 60 min at room temperature, and washed 3 times for 5 minutes with PBS. The cells were incubated with the primary antibody for 60 min and washed with PBS. The primary antibody used was anti-nestin (Rat-401, 1:100, Developmental Studies Hybridoma Bank, Iowa City, IA, USA), a marker of NSCs. The cells were subsequently incubated with Alexa Fluor546-labeled secondary antibodies (1:200, Molecular Probes, Eugene, OR, USA) for 30 min. The nuclei were counterstained with 4′,6-diamidine-2′-phenylindole dihydrochloride. Cells were viewed under a fluorescence microscope (BH-2; Nikon) and photos were analyzed with Photoshop Elements 12 (Adobe, CA, USA).

### Statistical analysis

Data are expressed as mean ± SEM. Differences between two groups were analyzed using the Student’s t-test or Welch’s t-test. Statistical significance was set at p < 0.05. All statistical analyses were performed using SPSS 26 Statistics Base (IBM Japan, Tokyo, Japan).

## Results

### Effects of culture period before HS on the numbers of proliferating NSCs after HS treatment

**Fig 2:**
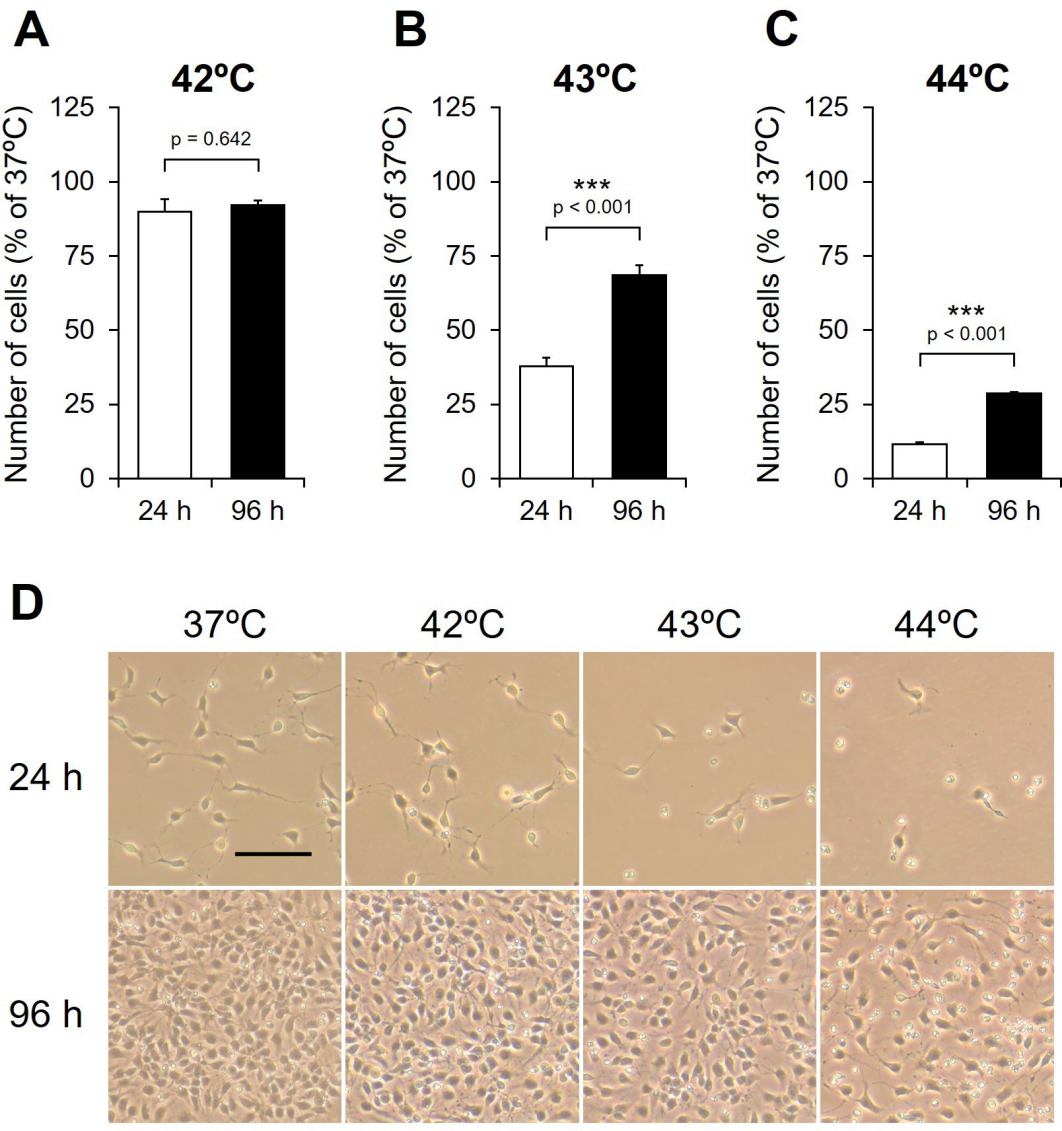
Effect of exposure to heat shock on the numbers of NSCs. NSCs were exposed to heat shock (HS) at (A) 42°C, (B) 43°C, and (C) 44°C for 20 min. (A-C) Numbers of adherent cells, 24 h after HS performed after culture at 37°C for 24 h (day 0; white) and 96 h (day 3; black). The values are presented as mean ± SEM percentages of controls (n = 6). ***p < 0.001. (D) Phase-contrast micrographs of 37°C cells and HS-exposed cells, 24 h after HS was performed and after culture at 37°C for 24 h (day 0; upper) and 96 h (day 3; lower).

To determine the effects of HS on the number of NSCs, the cells were exposed to HS for 20 min at 42°C, 43°C, or 44°C after culture at 37°C for 24 and 96 h (Fig 1B), and the number of adherent cells was counted and compared to number of cells exposed to 37°C. The ratios of adherent cells exposed to HS after 24 h decreased in a temperature-dependent manner (Fig 2A–2C), with the ratio being significantly lower after exposure to HS at 43°C (24 h, 37.88 ± 2.74%; 96 h, 68.54 ± 3.35%) and 44°C (24 h, 11.59 ± 0.56%; 96 h, 28.57 ± 0.64%) (Fig 2B and 2C), along with an increase in the number of floating cells (Fig 2D). The ratio of adherent cells exposed to HS at 42°C did not differ significantly between 24 h and 96 h (Fig 2A). In contrast, the ratios of adherent cells exposed to HS at 43°C and 44°C after culture at 37°C for 96 h were significantly higher than those exposed after culture for 24 h (43 and 44°C, p < 0.001) (Fig 2B and 2C). Cell morphology after HS was observed using phase-contrast microscopy. The edges of NSCs exposed to HS at 43°C and 44°C after culture at 37°C for 24 h were unclear and some cells lacked neurites (S1 Fig). In contrast, the edges of NSCs exposed to HS after culture at 37°C for 96 h were clear with all cells having neurites (S1 Fig). This indicates that cell morphology is dependent on both the temperature of HS and the culture period before exposure to HS. These results indicate that the culture period before HS differentially affects the remaining adherent cells of proliferating NSCs, even with HS exposure at the same temperature and duration.

### Effects of HS on expression of HSP genes in proliferating NSCs

**Fig 3:**
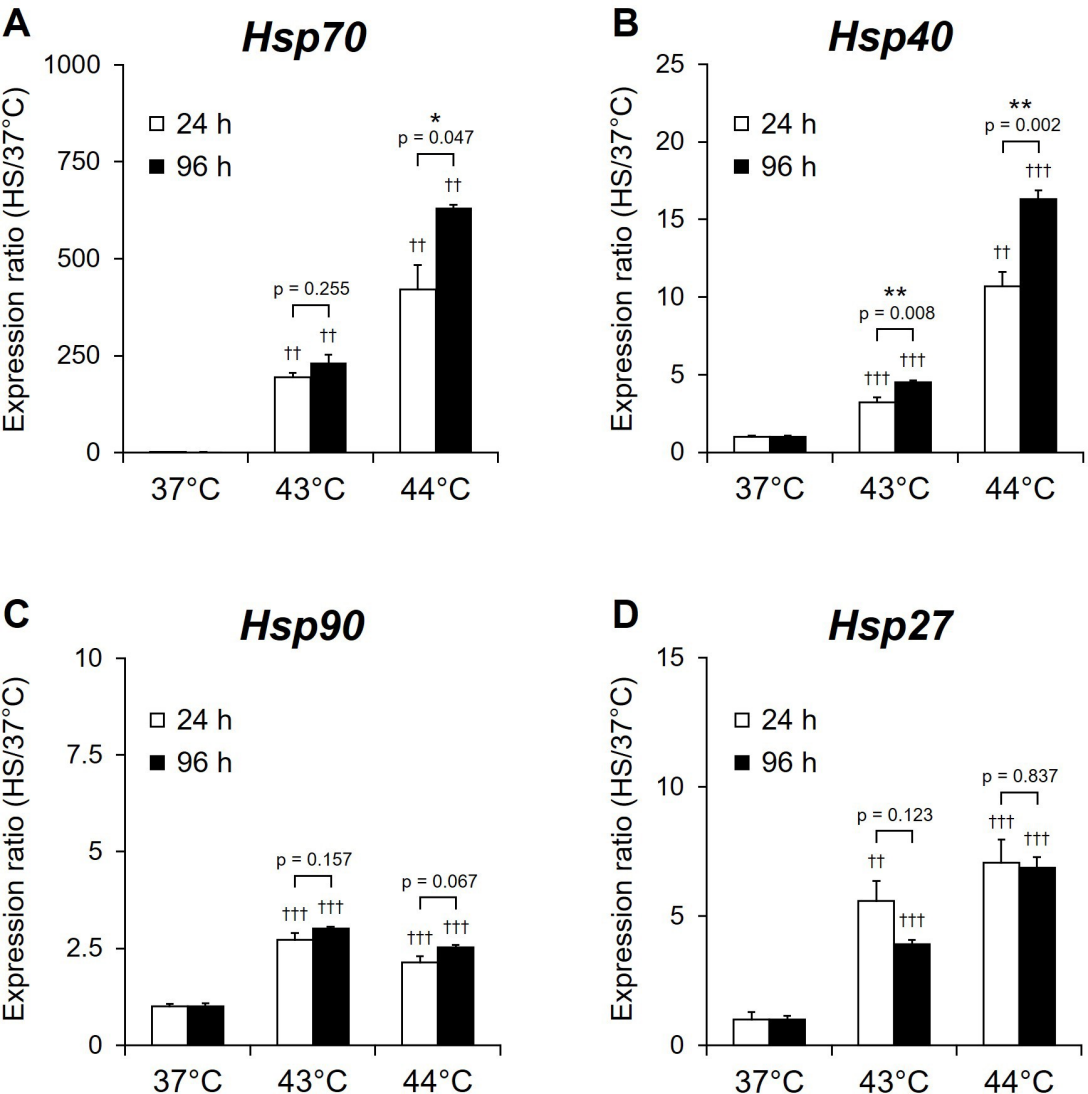
HSP gene expression 3 h after exposure of NSCs to heat shock. NSCs cultured for 24 h (day 0; white) or 96 h (day 3; black) under proliferation conditions were exposed to heat shock (HS) at 43°C or 44°C and were then allowed to recover for 3 h. The expression of (A) *Hsp70*, (B) *Hsp40*, (C) *Hsp90*, and (D) *Hsp27* was assayed by real-time RT-PCR and normalized by that of *RPS29*. Results are presented as means ± SEM (n = 4) (*P <0.05, **P <0.01 between 24 h and 96 h, ^††^P <0.01, ^†††^P <0.001 between 37°C and 43°C or 44°C).

To evaluate changes in HS responses in proliferating NSCs, the ratios of HSP gene expression 3 h after the HS were analyzed using real-time RT-PCR. The ratios of expression of all four HSP genes were significantly higher in NSCs exposed to HS at 43°C and 44°C (p < 0.01, p < 0.001) than in NSCs exposed to 37°C at both timepoints (24 and 96 h) (Fig 3A–3D). The increase in the ratios of *Hsp27*, *Hsp40*, and *Hsp70* was temperature-dependent (Fig 3A, 3B and 3D). HS at 24 and 96 h timepoints induced a 190- and 630-fold increase of Hsp70 expression, respectively. Expression of *Hsp70* when the HS was delivered at 96 h was significantly higher than at 24 h in the case of HS at 44°C and 43°C (p = 0.047 and p = 0.008, respectively) (Fig 3A). *Hsp40* expression was significantly increased after HS at 44°C for 96 h compared to that with 24°C (p = 0.002) (Fig 3B). These results demonstrate that the HSP expression increased with the extension of the culture period. In addition, these expression changes correlate with the change in the number of NSCs at these two time points in the culture (Fig 2B and 2C).

### Effects of HS on expression of apoptosis-related genes in proliferating NSCs

**Fig 4:**
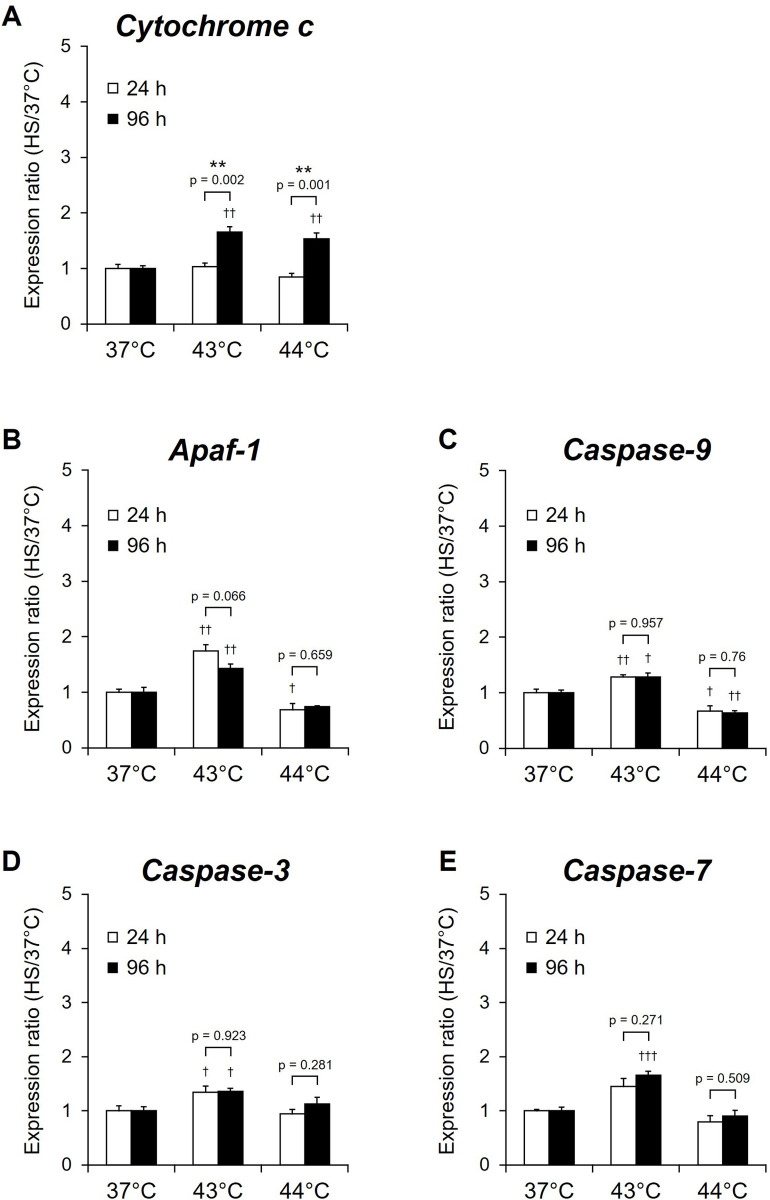
Expression of apoptosis-related genes 3 h after exposure of NSCs to heat shock. NSCs cultured for 24 h (day 0; white) or 96 h (day 3; black) under proliferation conditions were exposed to heat shock (HS) at 43°C or 44°C and were then allowed to recover for 3 h. The expression of (A) *cytochrome c*, (B) *Apaf-1*, (C) *caspase-9*, (D) *caspase-3* and (E) *caspase-7* was assayed by real-time RT-PCR and normalized by that of *RPS29*. Results are presented as means ± SEM (n = 4). (**P <0.01 between 24 h and 96 h, ^†^P <0.05, ^††^P <0.01, ^†††^P <0.001 between 37°C and 43°C or 44°C).

Expression of several HSPs induced by external stresses enhance cell survival by inhibiting the mitochondrial apoptotic pathways [35]. Therefore, to assess the effects on apoptotic signals of the increase of HSPs expression after HS in proliferating NSCs, the relative expression of the apoptosis-related genes *cytochrome c*, *Apaf-1*, *caspase-9*, *caspase-3*, and *caspase-7* were analyzed using real-time RT-PCR 3 h after cells were exposed to HS at 43°C and 44°C after culturing for 24 and 96 h. The relative expression of *Apaf-1* (24 h and 96 h; p < 0.01), *caspase-9* (24 h; p < 0.01, 96 h; p < 0.05), *caspase-3* (24 h and 96 h; p < 0.05), and *caspase-7* (96 h; p < 0.001) in NSCs exposed to HS at 43°C were significantly higher than those in cells exposed to 37°C (Fig 4B–4E). In contrast, the relative expression of *Apaf-1* (24 h; p < 0.05) and *caspase-9* (24 h; p < 0.05, 96 h; p < 0.01) was significantly lower than in the cells exposed to 37°C (Fig 4B and 4C). The relative expression of *caspase-9*, *Apaf-1*, *caspase-3*, and *caspase-7* did not differ significantly in cells exposed to the same temperature but at different timepoints (24 and 96 h) (Fig 4B–4E); however, *cytochrome c* expression was significantly higher than those exposed after culture for 24 h (43°C: p = 0.002; 44°C: p = 0.001) (Fig 4A).

### Regulations of cell viability and caspase activity in proliferating NSCs after HS

**Fig 5:**
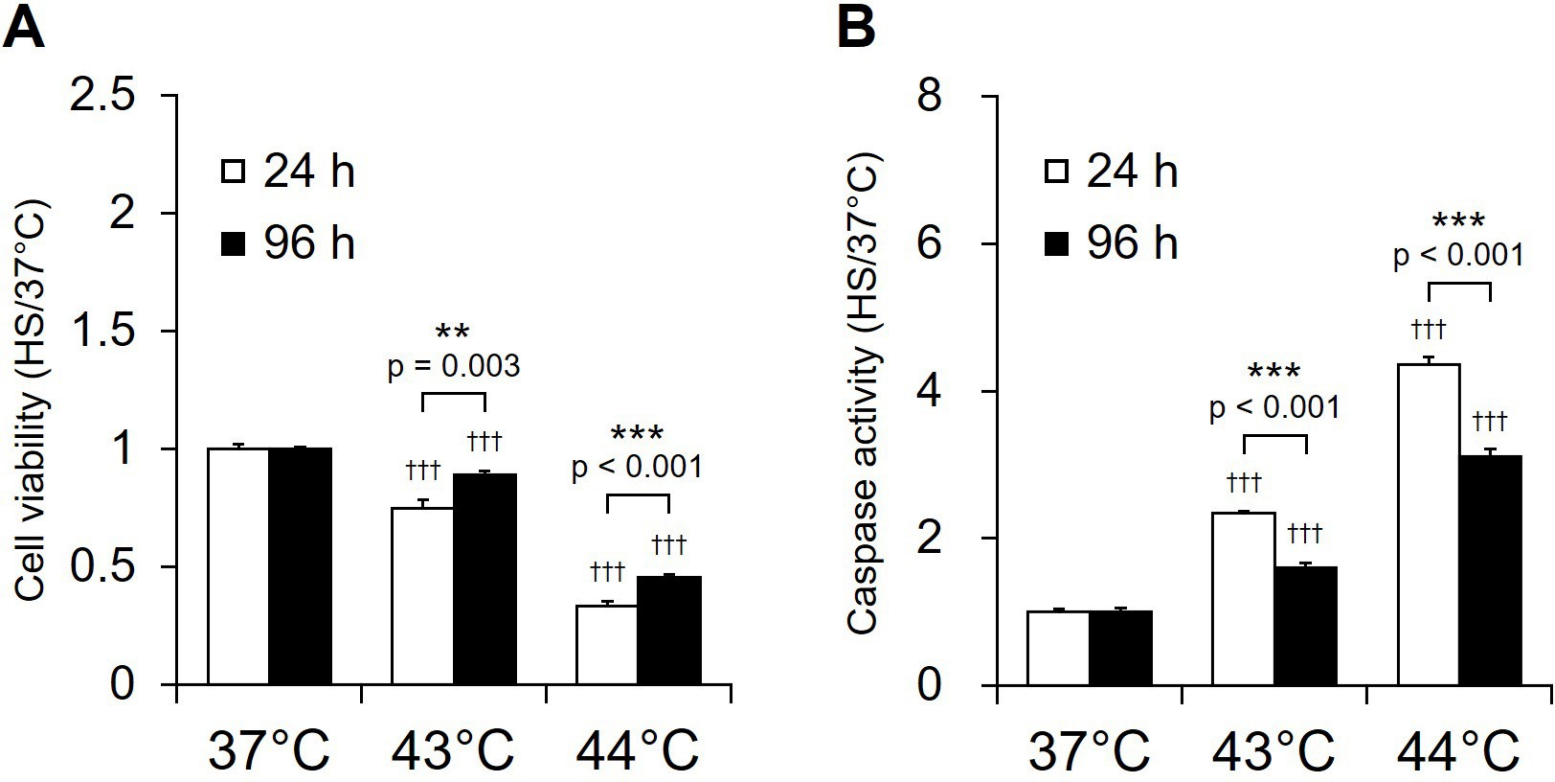
Caspase activities of NSCs exposed to heat shock. (A) Cell viability was measured 3 h after exposure to heat shock (HS) using CellTiter Glo reagent (Promega), with results expressed as mean ± SEM (n = 8) ratios to the 37°C sample. (**P <0.01 for comparisons between cells exposed to HS after culture for 24 h (day 0; white) and 96 h (day 3; black); ^†††^P <0.01 for comparisons between 37°C and 43°C or 44°C). (B) Caspase activity was measured 3 h after exposure to heat shock (HS) using the caspase-Glo 3/7 reagent (Promega) and normalized to cell viability measured using CellTiter Glo reagent (Promega), with results expressed as mean ± SEM (n = 8) ratios to the 37°C sample. The values are presented as the in the graph. (**P <0.01 for comparisons between cells exposed to HS after culture for 24 h (day 0; white) and 96 h (day 3; black); ^†††^P <0.01 for comparisons between 37°C and 43°C or 44°C).

To examine the factors leading to changes in cell number between 24 h and 96 h in proliferating NSCs after exposure to HS, cell viability assay and caspase activity assay, defined as the ratio of caspase 3/7 activity/cell viability, were performed. Cell viability was assessed at 24 h after HS, the same time period in which cell counts were performed. Cell viability after HS at 44°C was maintained for 3 h, but gradually decreased until 12 h (S2A Fig), and Caspase 3/7 activities increased within 20 min after HS at 44°C for 20 min and were maintained until 12 h (S2B Fig). Therefore, caspase activity in NSCs was analyzed 3 h after HS. After recovery by incubation at 37°C for 24 h after HS, cell viability of cells exposed to HS at 43°C and 44°C after culture at 37°C for 96 h were significantly higher than those exposed after culture for 24 h (43°C: p = 0.003; 44°C: p = 0.001) (Fig 5A). Caspase activity was significantly lower in cells exposed to HS at 43°C and 44°C after 96 h than after 24 h (43 and 44°C; p < 0.001) (Fig 5B). These results suggest that extension of the culture period prior to HS could improve cell viability after HS by inducing an increase in HSP expression and suppressing the increase in caspase activity.

### Maintenance of NSCs marker expressions in NSCs in culture

**Fig 6:**
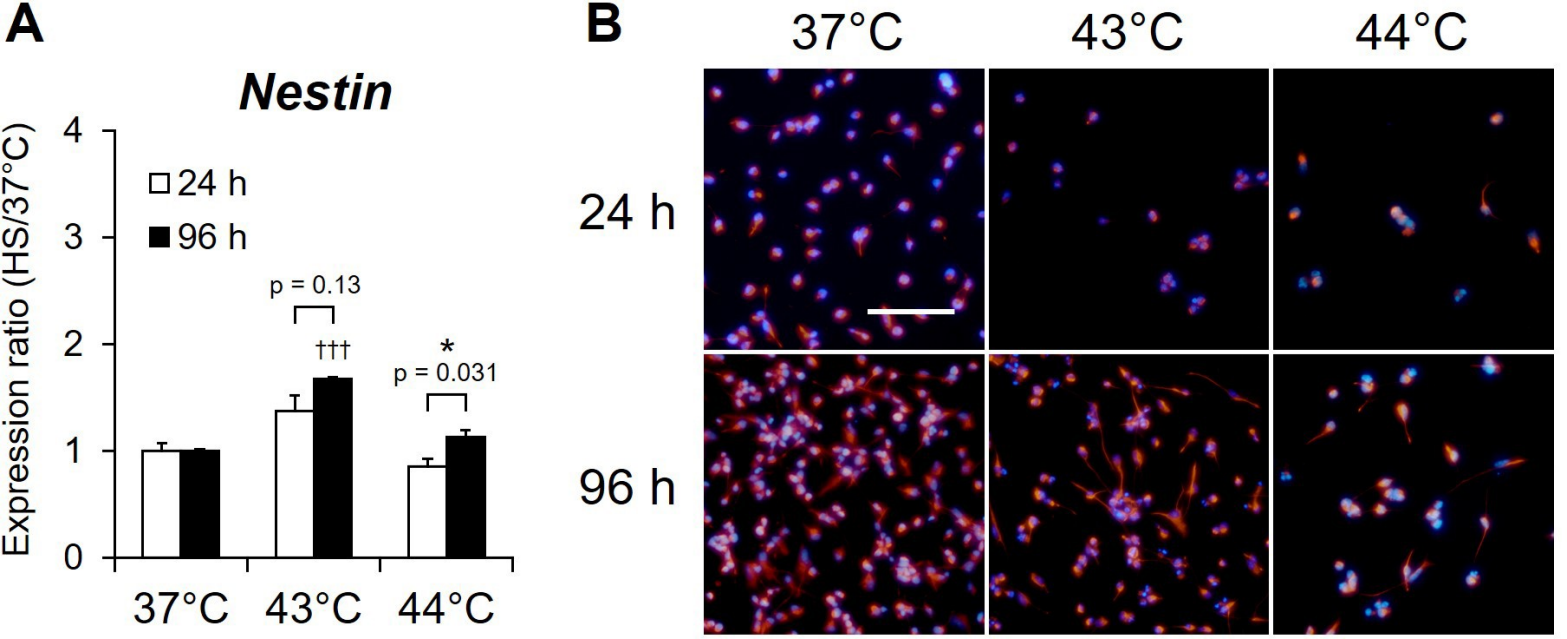
Expressions of the gene and the protein of the NSC marker after heat shock. (A) NSCs cultured for 24 h (day 0; white) or 96 h (day 3; black) under proliferation conditions were exposed to heat shock (HS) at 43°C or 44°C and were then allowed to recover for 3 h. The expression of *Nestin* was assayed by real-time RT-PCR and normalized by that of *RPS29*. Results are presented as means ± SEM (n = 4). (*P <0.05 between 24 h and 96 h, †††P <0.001 between 37°C and 43°C or 44°C). (B) NSCs cultured for 24 h (day 0; white) or 96 h (day 3; black) under proliferation conditions were exposed to heat shock (HS) at 43°C or 44°C and were then allowed to recover for 24 h. Immunofluorescence staining was performed on adherent cells after HS by using Nestin antibody. Fluorescence microscope images of Nestin (red) with DAPI counterstaining for nuclei (blue). Bars: 100 μm.

To evaluate the maintenance of the neural stem cells properties, the expression of NSC markers was evaluated using real-time RT-PCR analysis and immunofluorescence staining in NSCs at 3 h after at 37°C incubation or at 24 after 43°C and 44°C HS treatment. Real-time RT-PCR analysis indicated that the nestin gene, a marker that is specifically expressed in NSCs, was expressed in NSCs after HS at 37°C, 43°C, and 44°C for both 24 h and 96 h under proliferative conditions, and its expression at 96 h was significantly increased compared to that at 24 h (44°C: p = 0.031) (Fig 6A). Immunofluorescence staining demonstrated that NSCs after HS at 37°C were positive for nestin at both 24 h and 96 h under proliferative conditions, and similar results were observed for adherent cells after HS at 43°C and 44°C (Fig 6B). These results indicate that NSCs in culture maintain their properties as NSCs even after HS exposure and are preserved as NSCs although the culture period is extended.

## Discussion

We have previously shown that HS exposure above 43°C for 20 min before seeding in culture dishes inhibited the proliferation of ES cell-derived mouse NSCs [32]; however, the effect of the culture period prior to exposure to HS on the proliferating NSCs during culture under proliferative conditions was unknown. The current study shows that the culture period of proliferating homogeneous NSCs prior to HS exposure, even at the same temperature and for the same duration, profoundly affects the HS response, including the expression of HSP genes. Although HS above 43°C reduced the number of NSCs, the extension of the culture period prior to exposure to HS caused a relative increase in HSP expression, which resulted in a decrease in caspase activity and suppression of decreased cell viability. Several HSP genes whose expression is induced by HS help to limit the damage caused by stress and modulate apoptosis by different pathways [2]. Moreover, the relative expression levels of HS-induced HSPs are correlated with cell viability [5, 36–38]. The results presented in this study demonstrate that different HS conditions induce distinct HSP expression profiles, which effect the cell viability of a homogeneous population of proliferating NSCs cultured in proliferative conditions.

HSPs function as molecular chaperones, which prevent the aggregation of denatured proteins and promote protein refolding, and they also play a role in the primary resistance machinery to protect vulnerable cells [39] under both stress [40] and physiological [41] conditions. Hsp70 and Hsp27 have been reported to be the most strongly induced HSPs after stress, and they exert powerful neuroprotective effects [40, 42, 43]. Hsp40 also functions in the refolding of heat-denatured proteins by collaborating with Hsp70 [44]. Hsp90 is one of the most abundant proteins in cells in physiological conditions [45], with a function similar to that of Hsp70 and Hsp27 [46, 47]. Hsp27 and Hsp90 are also involved in regulating the cytoskeleton of neural cells; Hsp27 stabilizes major components of the cytoskeleton, including neurofilaments, actin, and microtubules [48] and is involved in neurite growth and/or axonal regeneration [49]. Hsp90 protects tubulin against thermal denaturation and maintains it in a state compatible with microtubule polymerization [50]. In the current study, we show that the soma and neurites morphology and the HSPs expression profile of NSCs exposed to HS at 43°C and 44°C were maintained by extension of the culture period prior to exposure to HS. Therefore, increased HSP expression may be protective in proliferating NSCs, maybe due to their function as molecular chaperones that refold denatured proteins, favoring cell viability.

We hypothesized that extension of the culture period before HS would not only affect HSP expression, but also the expression of apoptotic genes. In the mitochondrial apoptotic pathway, the leakage of cytochrome c from mitochondria activated of a downstream signal transduction cascade that includes complex formation by Apaf-1, recruitment of caspase 9, and activation of caspases 3 and 7, finally triggering apoptosis [35]. Hsp70 inhibits the release of cytochrome c from mitochondria [51] and together with Hsp90 interferes with Apaf-1 activity [51–53]. Hsp27 has also been reported to inhibit the release of cytochrome c [54, 55]. Inhibition of cytochrome c and Apaf-1 prevents the downstream activation of caspase 9, inhibiting apoptosis [51]. In the current study, there were no differences in the expression of apoptosis-related genes between 24 h and 96 h after HS except for *cytochrome c*. The absence of difference in HSP expression at 44°C compared to 37°C suggested that an increase in HSP expression is involved in the suppression of apoptosis-related genes. Because there were no changes in apoptosis-related genes during the culture period, we hypothesized that caspase 3/7 activity, which is located downstream of the apoptotic signaling pathway, and cell viability might be altered. Indeed, we found that caspase 3/7 activities were further inhibited by extension of the culture period; in contrast, cell viability improved. Altogether, our results suggest that expression of HSPs in NSCs induced by extension of the culture period before HS may have resulted in an anti-apoptotic effect by inhibiting the apoptotic signaling.

The survival of NS/PCs with mitotic capability has been reported to be affected by the extracellular matrix (ECM), which regulates the HS response and cell differentiation [56, 57]. Laminin, the main component of Matrigel^®^ Matrix, has been reported to maintain the stem cell properties of hippocampal NPCs, thereby enhancing their survival [58]. In addition, NS/PC differentiation caused a reduced HS response and suppression of Hsp70 expression, resulting in a high risk of vulnerability to stress [56]. In the current study, NSCs were positive for the NSC marker nestin at both the gene and protein level (Fig 6) and maintained the properties of NSCs with proliferative capacity in culture conditions using Matrigel^®^ matrix. These results indicate that maintaining properties under proliferative conditions and enhancing the HS response by extension of the culture period may modulate the survival of NSCs after exposure to severe HS stress.

In conclusion, the results of the present study demonstrate that mouse ES-derived NSCs under proliferative conditions show altered HSP expression and cell viability due to changes in the HS response and the properties of NSCs with the extension of the culture period prior to the HS. To the best of our knowledge, this is the first report to demonstrate that changes in the effects of HS on homogeneous NSCs occur in monolayer cultures under proliferative conditions. Stem cell therapies for regenerative medicine require to improve the viability of NS/NP cells after transplantation. The viability of transplanted cells may depend on the environment surrounding the foci as well as the condition of the donor cells [59]. Therefore, we believe that regulating the environment of NSCs before and after transplantation, including during culture, is critical for improving the viability of NSCs. Although the conditions in the current study were severe HS causing cell death, moderate HS could be a useful intervention to enhance cell survival, similar to what occurs in other neural cells. Studying HS conditions that are beneficial for the survival of stem cell-derived NSCs in culture or after transplantation may provide important insights to improve cell therapy.

## Supporting information

S1 Fig Cell morphology of NSCs after heat shock at 24 h and 96 h. Phase-contrast micrographs of control cells (left) and HS-exposed cells (43°C: middle, 44°C: right), 24 h after HS performed after culture at 37°C for 24 h (day 0; upper) and 96 h (day 3; lower).
(TIF)

S2 Fig Cell viabilities and caspase activities of NSCs 0–12 h after exposure to heat shock (HS). (A) Viability of NSCs 0–12 h after HS was assessed using CellTiter Glo reagent (Promega), with the results presented as the means ± SEM (n = 3). **P <0.01 for comparisons between control cells (circles) and cells exposed to HS at 44°C (triangles). Control cells were cultured in the 5% CO_2_ incubator at 37°C. (B) Caspase activities of NSCs 0–12 h after HS. Caspase activity of NSCs 0–12 h after HS was assessed using Caspase-Glo 3/7 reagent (Promega), normalized to cell viability using CellTiter Glo reagent (Promega). The results are presented as the means ± SEM (n = 3). **P <0.01 for comparisons between control cells (white) and cells exposed to HS at 44°C (black). Control cells were cultured in the 5% CO_2_ incubator at 37°C.
(TIF)

S1 Table Sequences of primer pairs used in quantitative real-time PCR. (DOCX)
