# Supplementary figures and images for: Heat shock response enhanced by cell culture treatment in mouse embryonic stem cell-derived proliferating neural stem cells
Source: PLoS One. 2021 Apr 14;16(4):e0249954. doi: 10.1371/journal.pone.0249954 (PMC8046196; doi:10.1371/journal.pone.0249954)

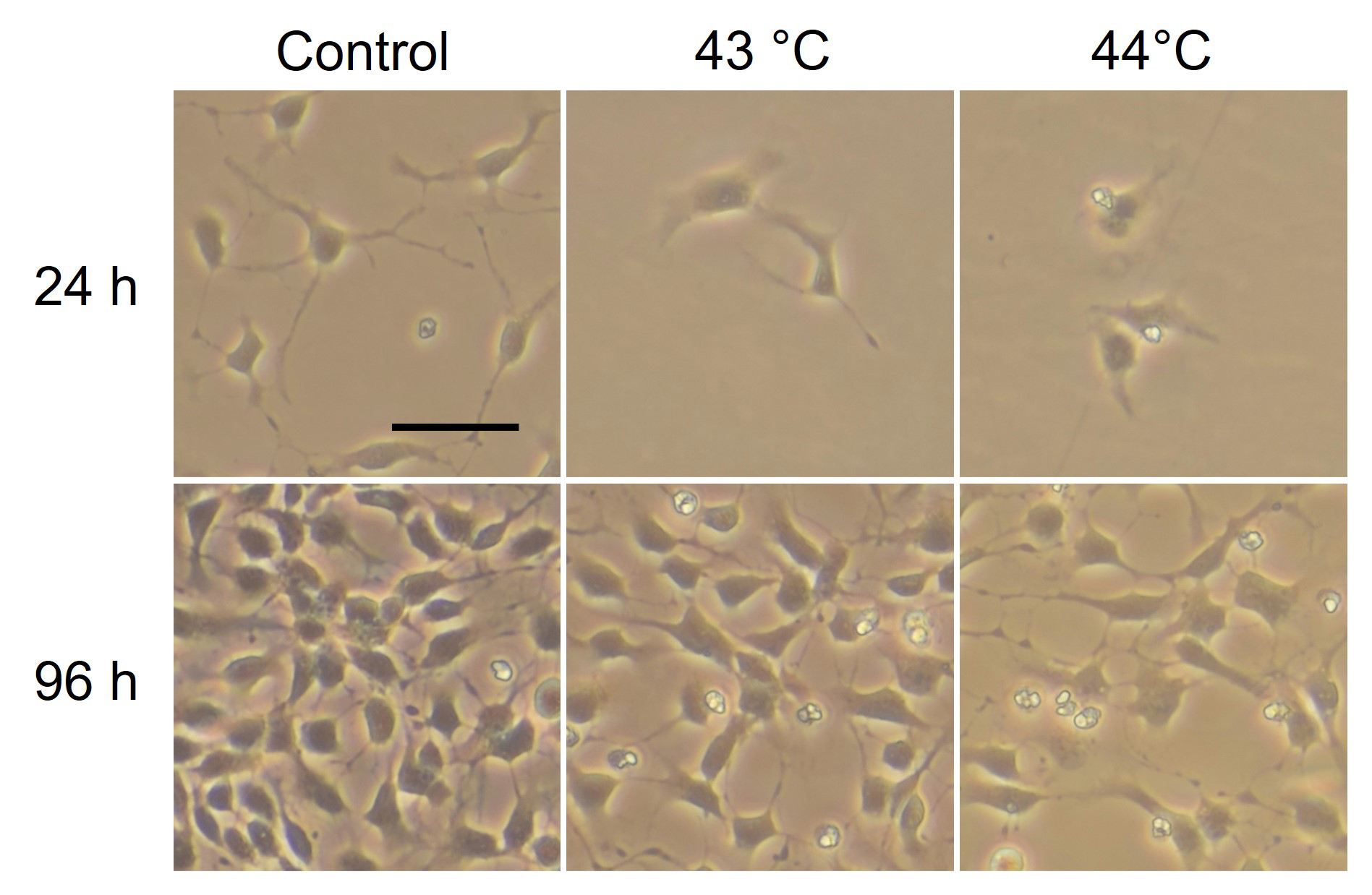

Supplement: S1 Fig — Phase-contrast micrographs of control cells (left) and HS-exposed cells (43°C: middle, 44°C: right), 24 h after HS performed after culture at 37°C for 24 h (day 0; upper) and 96 h (day 3; lower). (TIF) [file pone.0249954.s001.tif]

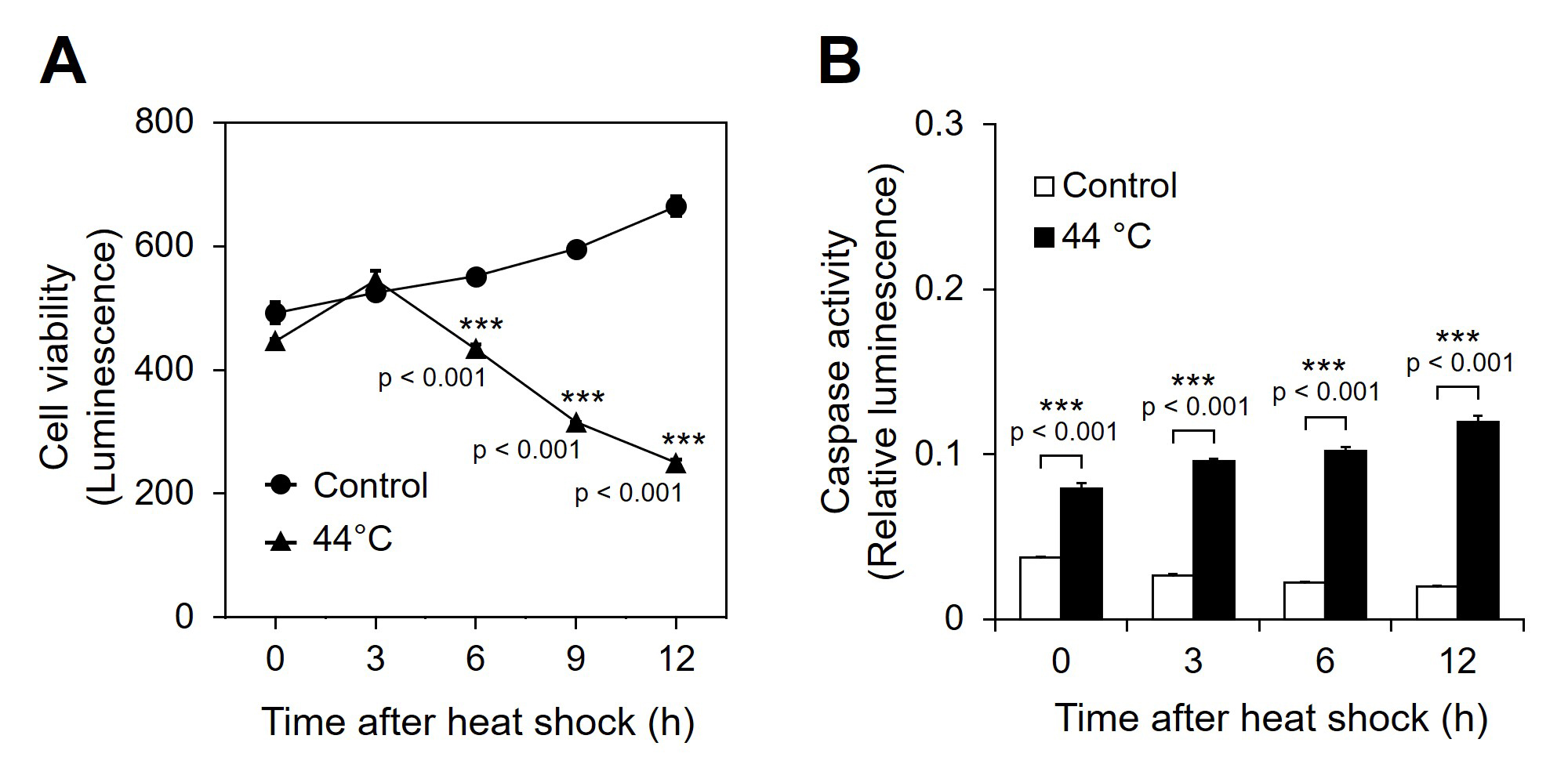

Supplement: S2 Fig — (A) Viability of NSCs 0–12 h after HS was assessed using CellTiter Glo reagent (Promega), with the results presented as the means ± SEM (n = 3). **P <0.01 for comparisons between control cells (circles) and cells exposed to HS at 44°C (triangles). Control cells were cultured in the 5% CO2 incubator at 37°C. (B) Caspase activities of NSCs 0–12 h after HS. Caspase activity of NSCs 0–12 h after HS was assessed using Caspase-Glo 3/7 reagent (Promega), normalized to cell viability using CellTiter Glo reagent (Promega). The results are presented as the means ± SEM (n = 3). **P <0.01 for comparisons between control cells (white) and cells exposed to HS at 44°C (black). Control cells were cultured in the 5% CO2 incubator at 37°C. (TIF) [file pone.0249954.s002.tif]
