# Supplementary material for: Heat shock response enhanced by cell culture treatment in mouse embryonic stem cell-derived proliferating neural stem cells
Source: PLoS One. 2021 Apr 14;16(4):e0249954. doi: 10.1371/journal.pone.0249954 (PMC8046196; doi:10.1371/journal.pone.0249954)
Supplement: S1 Table — (DOCX) [file pone.0249954.s003.docx]

**S1 Table. Sequences of primer pairs used in quantitative real-time PCR.**
